# Ultrafast spectroscopy reveals singlet fission, ionization and excimer formation in perylene film

**DOI:** 10.1038/s41598-021-83791-z

**Published:** 2021-03-04

**Authors:** Wenjun Ni, Licheng Sun, Gagik G. Gurzadyan

**Affiliations:** 1 State Key Laboratory of Fine Chemicals, Institute of Artificial Photosynthesis, Dalian University of Technology, Dalian, 116024 People’s Republic of China; 2 Department of Chemistry, School of Engineering Sciences in Chemistry, Biotechnology and Health, KTH Royal Institute of Technology, 10044 Stockholm, Sweden

**Keywords:** Optics and photonics, Photochemistry, Physical chemistry, Ultrafast photonics

## Abstract

Singlet exciton fission (SF) is a spin-allowed process whereby two triplet excitons are created from one singlet exciton. This phenomenon can offset UV photon energy losses and enhance the overall efficiency in photovoltaic devices. For this purpose, it requires photostable commercially available SF materials. Excited state dynamics in pure perylene film, ease of commercial production, is studied by time-resolved fluorescence and femtosecond transient absorption techniques under different photoexcitation energies. In film, polycrystalline regions contain perylene in H-type aggregate form. SF takes place from higher excited states of these aggregates in ultrafast time scale < 30 fs, reaching a triplet formation quantum yield of 108%. Moreover, at λ_ex_ = 450 nm singlet fission was detected as a result of two-quantum absorption. Other competing relaxation channels are excimer (1 ps) and dimer radical cation formation (< 30 fs). Excimer radiatively relaxes within 19 ns and radical cation recombines in 3.2 ns. Besides, exciton self-trapping by crystal lattice distortions occurs within hundreds of picosecond. Our results highlight potential of simple-fabricated perylene films with similar properties as high-cost single crystal in SF based photovoltaic applications.

## Introduction

Singlet fission is a phenomenon that happens in selected systems where a singlet excited molecule shares its energy with a neighboring molecule in its ground state, both molecules forming a pair of triplet excitons. Triplet formation via SF is a spin-allowed process, in contrast to spin-forbidden intersystem crossing, which usually proceeds in much faster femtosecond/picosecond time scale. SF materials are supposed to boost efficiency of photovoltaic cells through novel mechanism^1–3^. On one hand, nonlinear optoelectronic process SF (the absorption of one photon generates two triplet exciton-hole pairs) can theoretically break the Shockly-Queisser limit of 34% for single-junction conventional photovoltaics (one photon produces at most one exciton). On the other hand, important part of solar radiation is emitted in blue or UV spectral regions; after relaxation to the reactive state most part of excitation is transferred to heat, i.e. is lost. However, designing and synthesizing new SF molecular systems for real applications is attractive and quite challenging. SF studies are mostly focused on acenes, e.g. tetracene, pentacene, rubrene and related systems: molecular crystals, films and covalently linked dimers.

Perylene and its derivatives aroused a considerable interest in SF field due to their attractive properties, i.e. high absorption coefficients, photostability; they are widely used in various photovoltaic applications. In these systems perylenediimides stand out as a result of easy chemical modification and better energetics for singlet fission^4–7^.

In α- and β-perylene crystals and dimers, highly efficient ultrafast SF formation was demonstrated^8–10^. Singlet and triplet states of perylene are located at 2.86 and 1.53 eV, respectively. Therefore in order to realize SF, i.e. to fulfill requirement E (S_1_) ≥ 2 E (T_1_)^11,12^ one needs to excite higher states. Thus, for pure perylene exists a threshold E = 3.06 eV for SF^13^. As it was demonstrated in α-perylene crystal and perylene dimeric system, SF proceeds directly from higher vibrational states of S_1_ and upper electronic singlet state S_2_ state bypassing the lowest S_1_ state in t < 100 fs^8,9^. To the best of our knowledge, the excited state dynamics was not studied yet in perylene films. Compared with single crystal, fabrication of low-cost films is much simpler and more attractive for further applications.

In this work, we have fabricated perylene films by thermal vacuum evaporation and applied the ultrafast time-resolved techniques to excavate the possibility of singlet fission. Moreover, evolution of competing processes, e.g. excimer, dimer radical cation and self-tapped excitons formation/recombination was tracked as well.

## Results and discussion

### Film characterization

**Figure 1:**
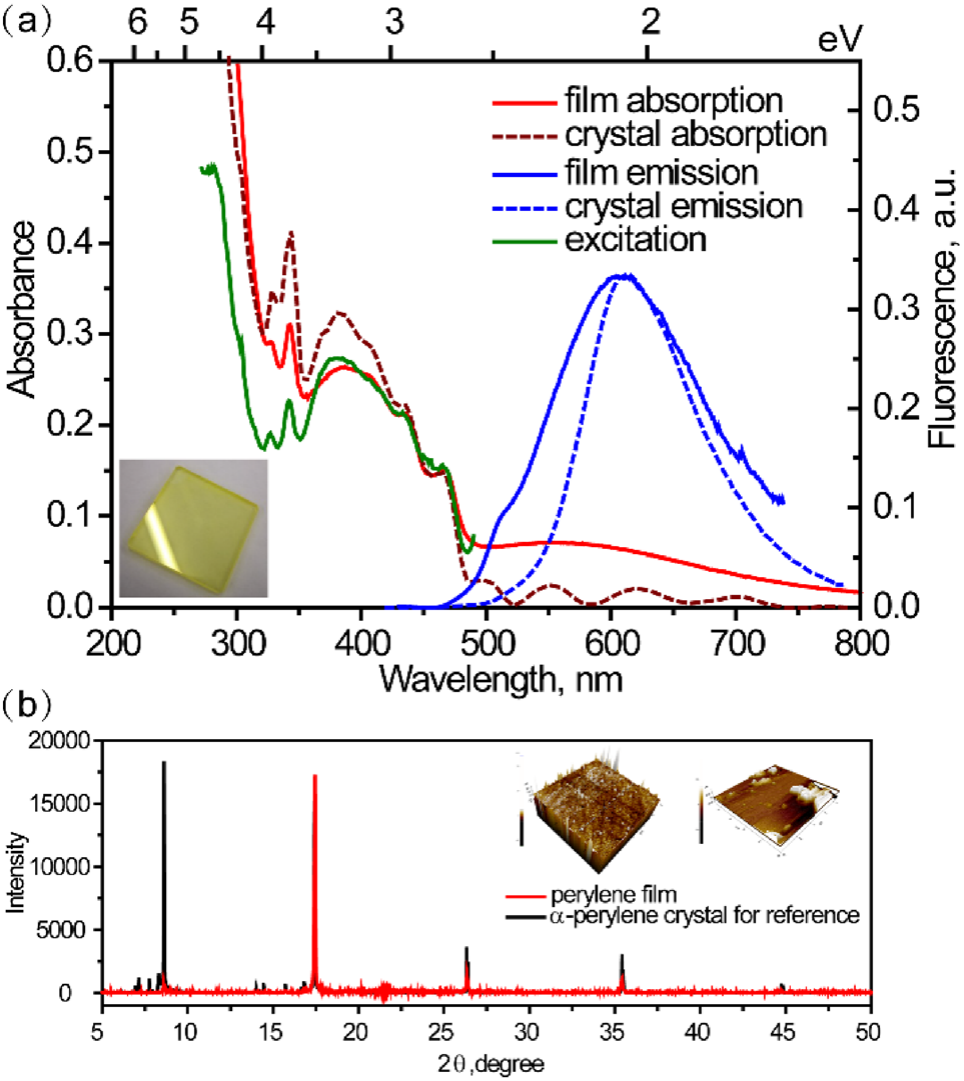
**(a)** Steady-state absorption, fluorescence and excitation spectra (solid curves) of perylene film (λ_ex_ = 390 nm, λ_em_ = 510 nm). Absorption and emission spectra (dotted curves) of α-perylene crystal are offered as reference (λ_ex_ = 400 nm)^8^. **(b)** XRD spectrum of perylene film (red curve) and of α-perylene bulk crystal (black curve). Insets show the AFM images of perylene film.

Morphological characterization of perylene film has been performed by use of scanning electron microscope (SEM) and atomic force microscopy (AFM) techniques. The cubic perylene nanoaggregates are clearly seen from SEM (Fig. S1a), distributed homogenously on the surface. As shown in Figure S1b, the particle size distribution was estimated in the range of 50–300 nm. AFM images (Fig. S2) also reveal the height of particles as 100–200 nm, which indicates that nanoaggregates have almost cubic shape. The thickness of film is estimated as 50–100 nm. In addition, to elucidate the real molecular packing in the film, X-ray diffraction (XRD) spectra of both perylene film and α-perylene single crystal have been recorded (Fig. 1b). All sharp lines position of our film spectrum in the 2θ range of 5°–50° match quite well with those of standard α-perylene crystal and the relative intensities are only slightly different. Therefore, we infer that here the molecular packing is mostly similar as in dimeric α-perylene single crystal. However, we cannot fully rule out existence of small fraction of monomeric perylene as well.

### Steady-state spectra

The absorption, fluorescence and excitation emission spectra of perylene film evaporated onto the fused silica plate are shown in Fig. 1a (solid curves). The film absorption spectrum shows a specific broad band between 320 and 500 nm with four humps at 468, 435, 342 and 328 nm. To resolve the transition assignments of each absorption band, Gaussian multipeak analysis was performed (Fig. S3) and the fit maxima were obtained (Table S1). The resulting constant energy spacing of 0.19 eV, corresponding to the C=C stretching energy, agrees well with the respective data for perylene solution (pink dotted curve in Fig. S3). Therefore, the absorption band between 2.66 and 3.62 eV corresponds to S_0_ → S_1_ transition^14^. As previously reported by Spano et al., the Coulombic coupling due to molecular aggregation can impact optical absorption spectra of perylene derivatives and lead to shift of the absorption maxima: red or blue shift for H- and J-aggregates, respectively^15,16^. For our perylene film, we found small red shift 0.02 eV compared with perylene solution (Fig. S3). It is indicative for existence of H-type aggregates in our film samples.

Another stronger absorption band below 320 nm is assigned to the S_0_ → S_2_ transition, supported by S_2_ absorption band at 250 nm in both perylene solution and crystal^8,9^. For comparison, absorption spectrum (red dotted curve) of α-perylene crystal is displayed as well. The shapes of the absorption spectra for film and crystal between 340 and 480 nm are almost the same, which points out that most molecules in film exist in highly ordered arrangement with dimeric H-type aggregated form. Existence of nanoaggregates and of α-form perylene conformation is further supported by SEM (Fig. S1) and XRD spectra (Fig. 1b), respectively. Additionally, there is a broad unstructured band ranging from 500 to 800 nm, which can be assigned to the light scattering of nano or microcrystals in the film^17^. Ishino et al. investigated the nanocrystals of α-perylene with size from 74 to 318 nm^18^. The phenomenon of size-dependent redshift and tail towards longer wavelengths were described in terms of light scattering and were supported by theoretical calculations^18^. The above shape of the absorption tail is indicative for existence of large amount of nanocrystals of various sizes, in agreement with our SEM characterization of 50–300 nm nanoaggregates.

Fluorescence emission with maximum at 606 nm is due to excimer; it is blue shifted compared to dimeric systems (613 nm) or crystal (610 nm)^9,19^. Compared with the reference fluorescence of crystal (blue dotted curve), the shoulder at about 500 nm can be ascribed to perylene monomer emission, supported by TCSPC results below. It should be noted that the discrepancy between absorption and fluorescence excitation spectra in the range of 250–310 nm is indicative of a photophysical process, which proceeds directly from the higher excitonic state bypassing S_1_ state, viz. singlet fission, which will be discussed below in detail.

### Time-resolved fluorescence spectra

**Figure 2:**
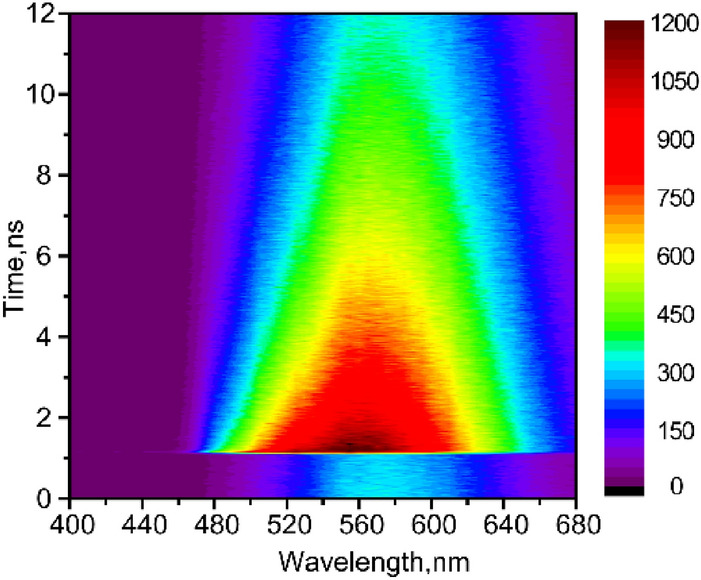
Time-resolved fluorescence map of perylene film, measured by TCSPC, λ_ex_ = 250 nm.

The fluorescence map of film in the wavelength range 400–680 nm was first collected by use of TCSPC: λ_ex_ = 250 nm (Fig. 2) and 380 nm (Fig. S4). Both maps exhibit the same kinetics, which indicates that excimer emission in film is independent on excitation wavelength. Kinetics at longer emission wavelengths (540–680 nm) exhibits single-exponential decay with time τ > 10 ns (Fig. S5). However, emission between 480 to 520 nm results in additional two shorter time components: τ_1_ ≤ 20 ps (limited by instrument response function) and the longer time component τ_2_ = 3.8–4.5 ns with varying amplitudes. The data at λ_ex_ = 380 nm were globally fit to three exponentials: resulting decay-associated spectra (DAS) are shown in Figure S4(b). The DAS contains three fluorescence spectra with maxima at 500 nm (0.06 ns), 520 nm (3.2 ns) and 590 nm (19 ns). The emission maximum at ~ 590 nm is in good agreement with excimer fluorescence in crystal and dimers^8, 20,21^. The spectrum with maximum at 520 nm (τ = 3.2 ns) is due to existence of certain amount of perylene monomers, which is consistent with τ = 4 ns for perylene in hexane^22^. The shortest time component (< 0.06 ns) results from the locally excited singlet state and hot excimer state, which was further quantified by up-conversion technique with higher time resolution.

**Figure 3:**
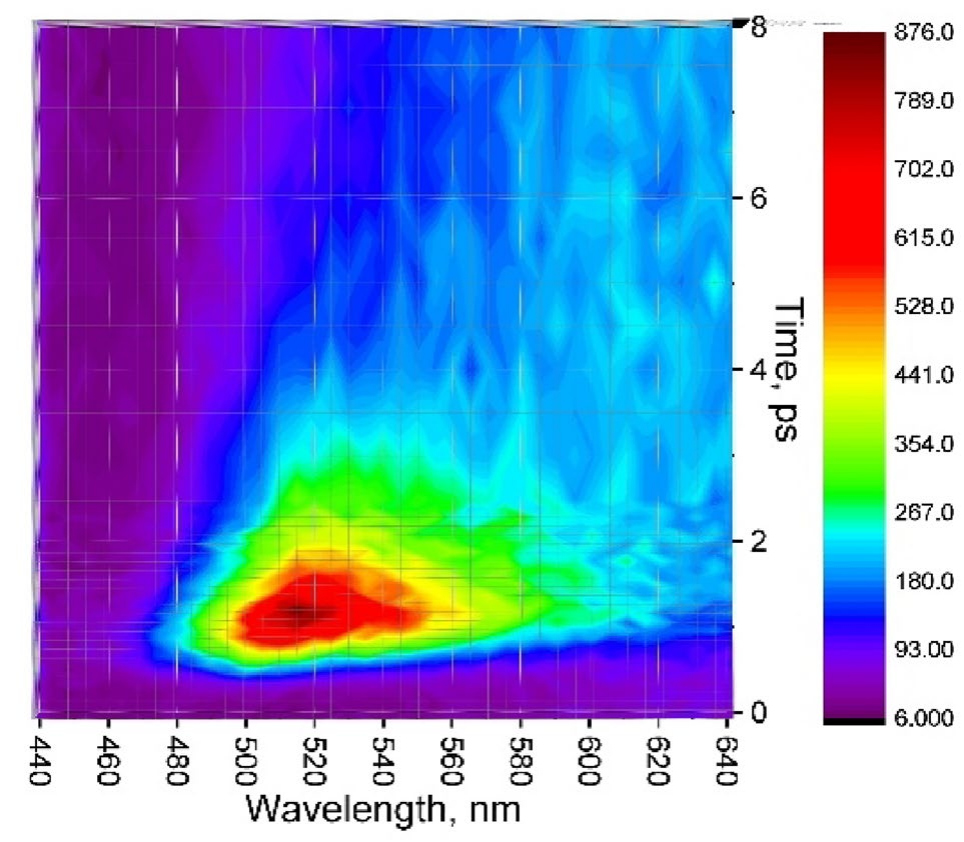
Up-converted fluorescence map of perylene film, λ_ex_ = 400 nm.

Figure 3 records fluorescence map at short time range 0–8 ps with 100 fs resolution. An ultrafast decay component of τ = 850 fs was observed in the range of 460–500 nm (Table S2). This emission band can be attributed to the quenched locally excited singlet state of perylene^9,21^. The other broad region, 500–560 nm, originates from hot excimer, in agreement with Refs.^23–25^. This hot excimer relaxes within 0.45–1 ps; compare with 0.5–3 ps in crystal^8^.

### Femtosecond transient absorption (fsTA) spectra

**Figure 4:**
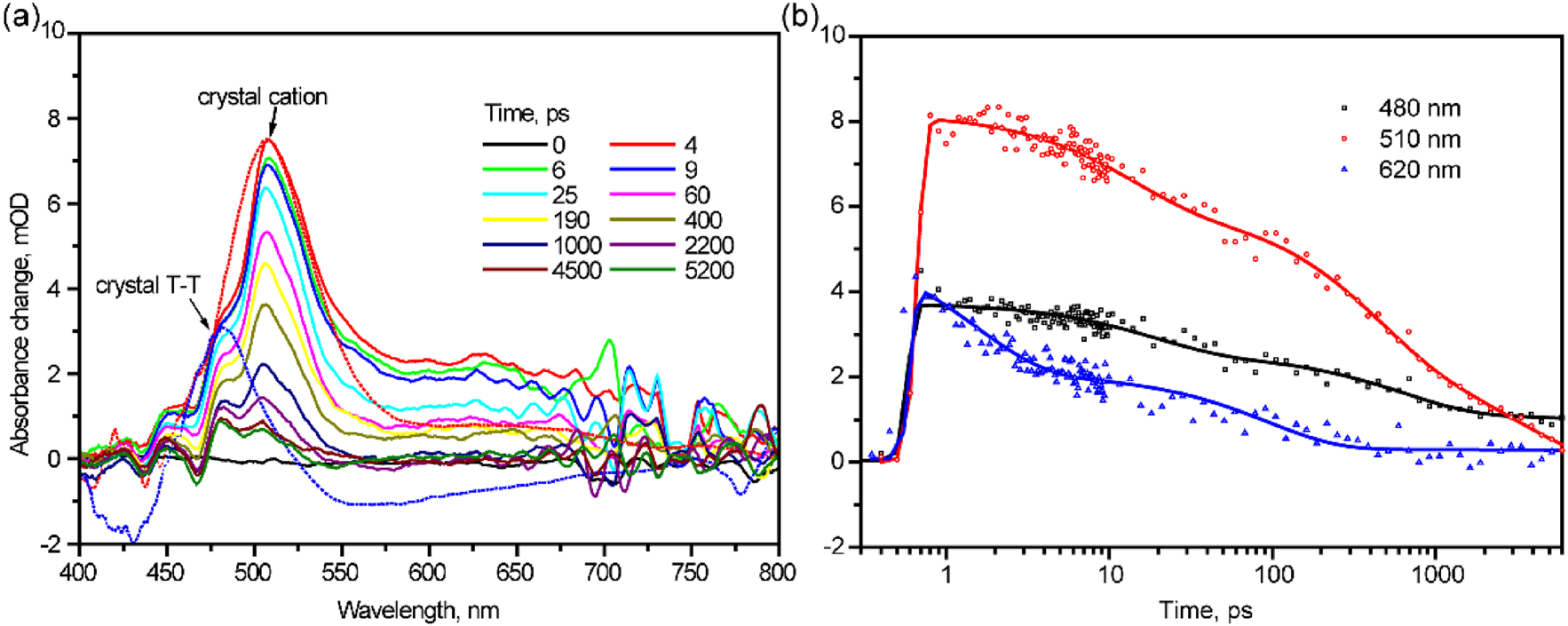
Femtosecond transient absorption spectra **(a)** and kinetics **(b)** in perylene film, λ_ex_ = 250 nm.

In order to study higher excited states relaxation processes in perylene film, fsTA measurements employing 250 nm excitation were made. The fsTA spectra (Fig. 4a) display a single positive band ranging from 440 to 550 nm and a weaker broad tail between 600 and 800 nm. The structured excited state absorption (ESA) band (440–550 nm) exhibits three maxima: 450, 480, 510 nm. Obviously, ESA at 510 nm (Fig. 4b) decays much faster than that at 480 nm, indicating that they belong to different transient species.

In entire TA spectra, we did not observe ground state bleaching (GSB) and stimulated emission (SE) signal, similar to the case of α-perylene crystal^8^. This can occur because of several reasons. First, the ESA is much stronger than GSB, e.g. the absorption cross section of the S_1_ → S_N_ transition in α-perylene crystal is 50 times larger than that of S_0_ → S_1_^8^. Free carriers which are generated during the laser excitation can strongly (orders of magnitude) enhance the absorption cross-section due to “infrared-activated vibrations” (IRAV)^26^. Second, bright excitons reside at the band-top in H-type aggregates, i.e. these states have high transition dipole moments. We excite molecules to band-top, and further excitons in ultrafast scale relax to the band-bottom, which has very weak transition dipole moment. Therefore, observed ESA is much stronger than the GSB^27^.

### Dimer radical cation formation

In order to identify the states/photoproducts produced from singlet excitons, we compare our spectra with known TA spectral features in α-perylene crystal (Fig. 4a, red dashed curve). The shape of TA curve of film follows the trace of crystal. In α-crystal this well-marked 510 nm ESA was the result of photoionization with formation of perylene dimer cation radicals. They were observed and studied in gas phase and liquid sulfuric acid^28,29^. This spectral feature was reported in different systems: perylene monomer in boric acid with λ_max_ = 508 nm^29^, perylene dimer in hexane and acetonitrile with λ_max_ = 520–600 nm^9,21,30^, in α-perylene crystal with λ_max_ = 503 nm^8^. Therefore, our observed ESA at 510 nm we assign to the perylene dimer radical cation (Per ^•+^).

In our films, ESA at 510 nm, rises instantaneously (< 30 fs) and decays triexponentially with τ_1_ = 13 ps (26%), τ_2_ = 410 ps (43%) and τ_3_ = 3.3 ns (31%). Here Per ^•+^ formation proceeds in < 30 fs and final recombination occurs within 3.3 ns. In the previous publications, Per ^•+^ lifetimes were studied in concentrated sulfuric acid t = 15 ps^31^, or 26 ps^32^, in boric acid glass t = 35 ps^31^, in freon glass at 77 K t = 100 ps^32^. Besides, they also mentioned that glass defects can also quench the lifetime of radicals. Note that in similar aromatic hydrocarbon molecules, e.g. biphenyl, naphthalene and anthracene, cation radicals decay even much faster, within 200 fs^33^. In contrary, changing pH from 2 to 7 results in increase of Per ^•+^ lifetime from picoseconds to microseconds^34^. The second lifetime in ESA decay (410 ps) we assign to self-trapping of excitons (see below).

### Triplet formation

The 480 nm shoulder of ESA band in film (Fig. 4a) is more pronounced than in α-perylene crystal (blue dash curve)^8^. This ESA band rises within the instrument response (< 30 fs) and subsequently decays with three time components: τ_1_ = 18 ps, τ_2_ = 650 ps and τ_3_ >  > 10 ns. This 480 nm TA band we assign to triplet–triplet absorption, in agreement with previous publications: 485 nm in perylene monomer^35–37^, 480–525 nm in perylene dimer^9^, 480 nm in α-perylene crystal^8^.

Well-known pathways of triplet formation are spin-forbidden intersystem crossing (ISC) S_1_ → T_1_, and spin-allowed SF S_1_ → T_1_ + T_1_. Because of large S_1_-T_1_ energy gap and planarity of the molecule, ISC in perylene is not efficient, ϕ_isc_ < 0.01^38^. However, in perylene film we have observed triplet TA at λ_ex_ = 250 nm, well above the energy threshold. Thus, we claim that it forms due to SF. The yield of singlet fission was calculated by using the methodology used in Refs.^7^,^39^. For this purpose we need to know the extinction coefficients of triplet–triplet absorption of perylene in film. Therefore, we have performed three different measurements: a) perylene triplet sensitization^7,40^ by using BODIPY or b) anthracene as a photosensitizer; c) comparison with a known molecule (or transient^39^): 3-bromoperylene. First two methods give large uncertainty (see Supplementary Information, pp. 9–14). Our estimation of the quantum yield of triplet formation based on the third method (3-bromoperylene) is ϕ_T_ = 108%, accordingly, ϕ_SF_ = 54%.

It was demonstrated that molecular packing has a strong effect on the yield of SF in organic semiconductors: slip-stacking favors delocalization of excitation and enhances exciton fission^41^. J-type tetracene aggregates lead to fast triplet formation by SF^42^. Quantum interference theoretical calculations show that in J or H type aggregates SF is more efficient^43^. Here perylene is organized as H-type aggregates and homogenous distributed in the film surface; it is favorable condition for SF. Moreover, shortening of triplet lifetime in our case is also a strong indication for existence of SF. Hence, we conclude that triplets form via SF between perylene aggregates within t < 30 fs at λ_ex_ = 250 nm. Note that competition between SF and excimer formation was studied in detail also in films containing various perylene diimides^6^. It was demonstrated that molecular packing due to introduction of bromine atoms is crucial in excitonic interactions.

Spin-obit charge-transfer enhanced intersystem crossing mechanism (SOCT-ISC) may also contribute in triplet formation. Intersystem crossing via a symmetry-breaking charge transfer in zinc dipyrrin complex was observed within 1–5 ps. In this case, real donor/acceptor interface is expected inside the complex^44^. However, in our film is less probable generating triplets via this mechanism. First, our film was constructed by use of pure perylene molecules. Strong charge transfer is not expected between two identical perylene molecules. Second, there are few molecular configuration and electronic coupling requirements for the charge transfer mechanism. Parameters that influence the efficiency of charge transfer enhanced intersystem crossing are the following: degree of charge transfer from donor to acceptor, the dihedral angle θ between their π systems, and the magnitude of electronic coupling between donor and acceptor^45^. Moreover, the efficiency is maximal at θ = 90°. In our previous work on multicomponent molecular crystal (perylene-TCNQ), we observed the triplet formation via charge transfer mechanism between donor perylene and acceptor TCNQ. Note that in that case perylene stays almost perpendicular to TCNQ^46^. In our present case with perylene film, the steady-state absorption is almost identical to α-perylene crystal. It indicates that our films are of H-type aggregated morphology with face-to-face molecular packing (as in the crystal), but not orthogonal. Even though, we cannot fully rule out contribution of charge transfer intermediate to enhancement of triplet states formation. It should also be mentioned that existence of dimer radical cation (as a result of ionization) cannot be involved in ISC process. In several publications with various systems it was clearly demonstrated that separated radical anions or cations do not lead to generation of triplets^47–49^.

Moreover, we have observed an ultrafast (< 30 fs) rise of the triplet transient, which is an additional argument for the triplet formation directly from upper excited states via SF and not through SOCT or radical cation.

In our case, perylene triplet TA band overlaps considerably with the absorption of dimer radical cation. The triplet TA did not decay at 6 ns, i.e. the longest delay time of our setup. However, in contrast with the α-perylene crystal (Fig. 4a, blue dashed curve), where the triplet TA remains at t = 1 ms, in film we did not see any remaining triplet transients at “negative times”. Note that negative times mean that the probe beam comes earlier than the pump beam. Because the repetition rate of our laser is 1 kHz, we can consider that the observed signal is generated by the previous pulse, i.e. the actual time delay between pump and probe pulses is 1 ms. In order to resolve our lifetime of triplet state in film, nanosecond flash photolysis technique was applied at λ_ex_ = 355 nm and we probe in the triplet–triplet transition range at λ_probe_ = 480 nm (Fig. S6). The lifetime of triplet state was obtained: τ = 85 ns.

Our obtained triplet lifetimes for perylene films are significantly shorter than in deoxygenated solution (4.8 ms)^35^. The shortening of the triplet state lifetime in films is a well-known phenomenon. It was demonstrated that the triplet lifetime of phthalocyanine films decreased from 75  to 11 µs compared with isolated molecules in solvent. This effect was explained in terms of electron and energy transfer between adjacent molecules^50^. Similar shortening of triplet lifetimes was demonstrated in hexathiophene films resulting from numerous defects and aggregates traps^51^. Triplet–triplet annihilation (TTA) was also shown to shorten triplet lifetimes, e.g. in carotene aggregates τ_T_ < 1 µs relative to 20 µs for monomeric molecule^52^. Therefore, shortening of triplet lifetime in perylene film is considered to be due to charge/energy transfer between perylene species or from perylene to the defect/trap states of quartz.

**Figure 5:**
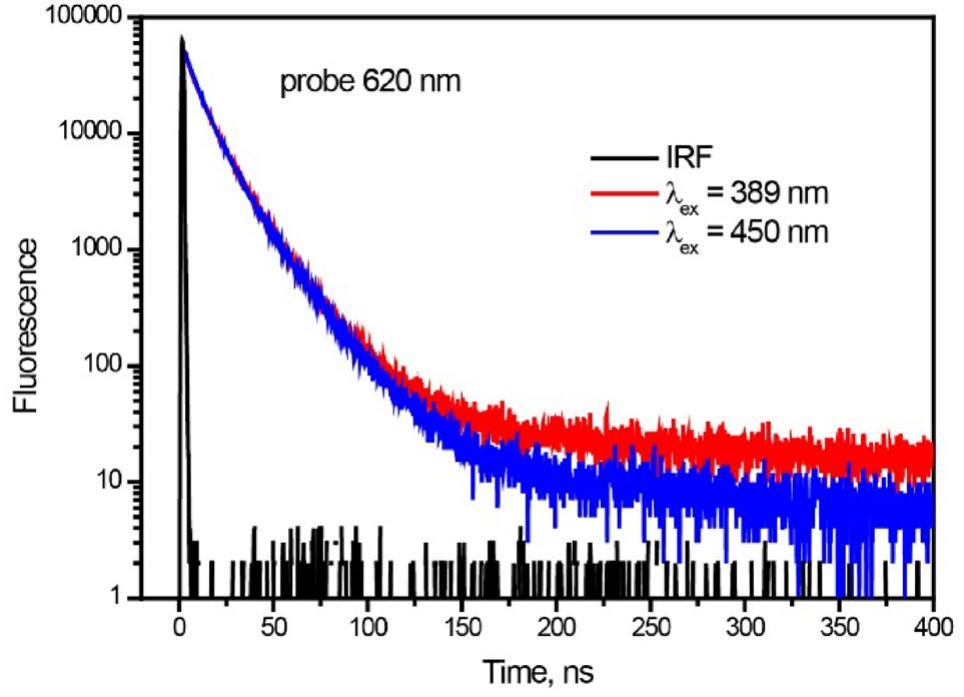
Time-resolved photoluminescence decay trace of perylene film ar 620 nm, λ_ex_ = 389 and 450 nm.

In our case, the shortening of triplet lifetime can also be due to TTA from neighboring triplet excited molecules, which are formed via SF. In film, there exists energy level matching: 2E(T_1_) > E(S_1_). Note that E(S_1_) = 2.86 eV, E(T_1_) = 1.53 eV. This condition is favorable that two triplet excitons can annihilate efficiently to form the S_1_ state, which could in turn produce delayed fluorescence. In order to proof this hypothesis, we have measured Time-resolved photoluminescence (PL) in the larger time window with λ_ex_ = 450 and 389 nm, i.e. below and above SF threshold (Fig. 5). At λ_ex_ = 450 nm, decay is monoexponential with τ = 17 ns, whereas at λ_ex_ = 389 nm kinetics exhibits two time components: τ_1_ = 17 ns, τ_2_ = 82 ns. The shorter lifetime (17 ns) is assigned to the excimer PL, which corresponds to above τ = 19 ns (Fig. S4b). The longer time 82 ns at λ_ex_ = 389 nm agrees well with the triplet lifetime 85 ns obtained from ns flash photolysis. It means that the longest PL at λ_ex_ = 389 nm can be assigned to delayed fluorescence due to TTA, which strongly supports our arguments on triplet formation via singlet fission.

### Two-quantum induced singlet fission

**Figure 6:**
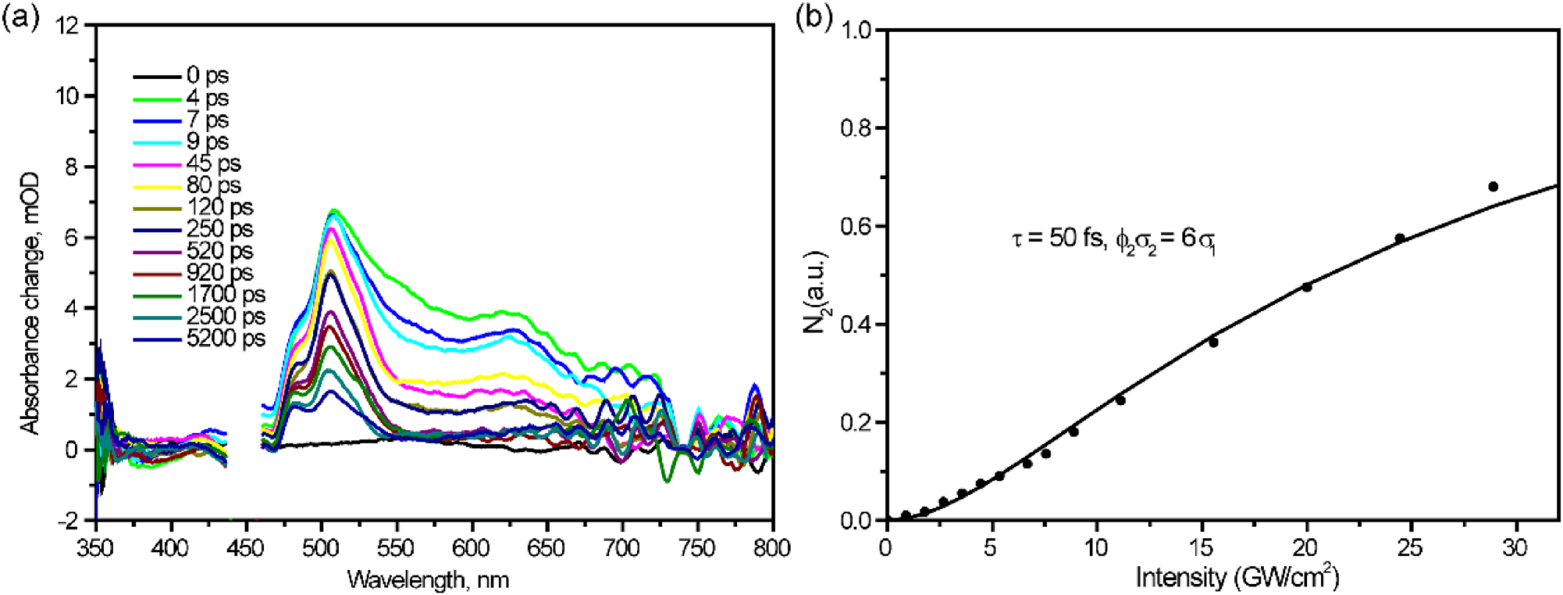
**(a)** fsTA spectra of perylene film atλ_ex_ = 450 nm. **(b)** Intensity dependence of the 480 nm transient (TT absorption) versus the laser excitation intensity.

The fsTA spectra at 450 nm excitation are shown in Fig. 6a and accordingly kinetics is presented in Table S3. Note that the excitation photon energy is below the threshold for SF. However, here triplet TA signal at 480 nm still dominates the whole spectrum. Moreover, the intensity dependence of the TA signal is obviously nonlinear. Similar to the case for perylene crystal^8^, we explain this nonlinear dependence in terms of consecutive two-quantum absorption (TQA) via intermediate state S_1_ (Scheme S1). The best fit is obtained for ϕ_2_σ_2_ = 6σ_1_, where σ_1_ and σ_2_ are absorption cross-sections from S_0_ and S_1_ states, respectively, and ϕ_2_ is the quantum yield of two-quantum photoproduct (Fig. 6b). The analytical formula for TQA and fit process is described in Supplementary Information. On the basis of the above considerations, we claim that singlet fission at λ_ex_ = 450 nm is due to TQA. Even though 450 nm two-quantum excitation leads to population of higher electronic states (5.5 eV) than upon direct 250 nm excitation (5 eV), SF in the former case proceeds slower: compare 170 fs with < 30 fs.

### Other photophysical processes

A closer look to the femtosecond transient absorption spectra depicts weak ESA around 700 nm, similar to singlet–singlet absorption of perylene monomer^19^. This band decays with τ = 1.0 ps (Fig. S7), which corresponds well to S_1_ fluorescence lifetime of perylene film (τ = 850 fs). Another weak broad band at λ_max_ = 620 nm is due to excimer, in agreement with our previous publications for perylene dimers and α-perylene crystal^8,9^. We cannot clearly resolve the formation time of excimer because of its strong overlap with ESA of self-trapped exciton. However, by use of time-resolved fluorescence, hot excimer relaxation time and excimer formation time were clearly resolved: 0.4–0.8 ps and 1 ps, respectively. In fact, with λ_ex_ = 380 nm (TCSPC) and λ_ex_ = 400 nm (up-conversion), we were exciting the S_1_ state. Accordingly, we resolved lifetimes of different states: the locally excited S_1_ state (850 fs); hot excimer (0.45–1 ps); relaxed excimer (19 ns), as well as the remaining perylene monomer (3.2 ns). In addition, by using pump–probe technique, when excited S_1_ (450 nm) and also S_2_ state (250 nm), we monitored formation of the dimer radical cation (510 nm) and triplet state (480 nm) with lifetimes 3.3 and 82 ns, respectively. Moreover, weak broad excimer ESA at about 620 nm with τ > 6 ns and S_1_ → S_N_ absorption at 700 nm with τ = 1 ps were also observed. Both TRPL and TA data give for S_1_ state lifetime t = 1 ps and for excimer lifetime t = 19 ns. Therefore both methods lead to reliable and consistent results.

In order to view the full relaxation picture, the fsTA data at λ_ex_ = 250 nm were globally fit, and the resulting Evolution-Associated Spectra (EAS) (Fig. S8) exhibit maxima at 480 nm (> 10 ns), 504 nm (3.2 ns), 515 and 550 nm (56 and 1.5 ps). The 504 nm band is assigned to dimer radical cation, in agreement with Ref.^28^,^29^. The longest time component (> > 10 ns) is triplet–triplet absorption. In addition, the broad EAS with the shorter time constant 56 ps is assigned to self-trapped exciton. Similar TA band in transient absorption microscopy with λ_ex_ = 680 nm was observed in β-perylene crystal and was assigned to self-trapping of free excitons (formation time 15 ps)^53^. Excitons were stabilized by self-induced crystal lattice distortion through phonon-exciton coupling. In α-perylene crystal with dimeric molecules in unit cell, exciton self-trapping runs with two time constants: < 100 fs and 2 ps^19,53^. Besides, self-trapped excitons were also observed in aggregated molecular nanostructures^54^. Interestingly, lifetime of self-trapped excitons formed after 250 nm excitation are longer than that formed after λ_ex_ = 450 nm. In addition, the shortest time constant 1.5 ps is a result of intramolecular vibrational redistribution (IVR) in S_1_ state, which was observed after excitation of S_2_ state.

**Figure 7:**
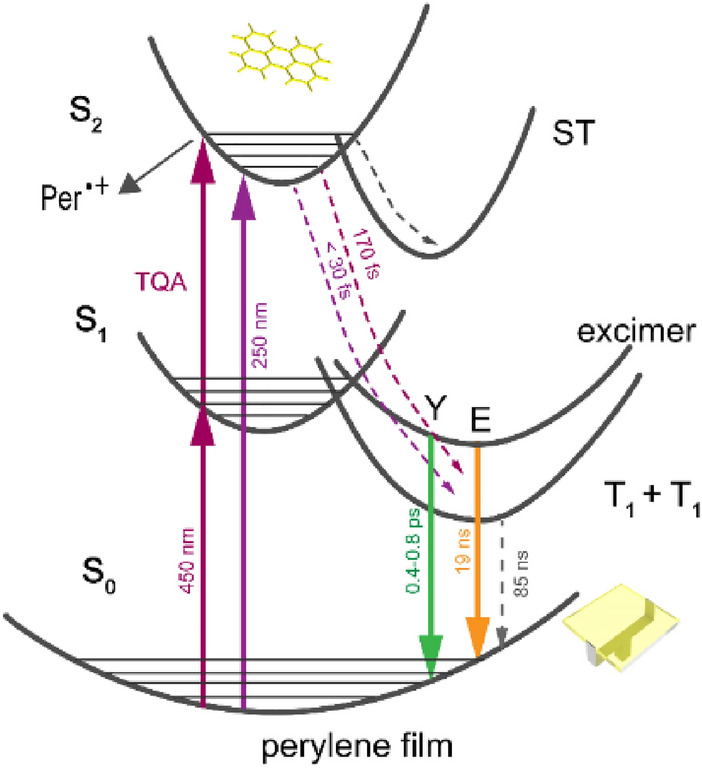
Photophysical pathways of perylene film at λ_ex_ = 450 and 250 nm. Per ^•+^ is perylene dimer radical cation. Y is hot excimer and E is relaxed excimer. T_1_ + T_1_ is the triplet pair state generated through singlet fission. ST is self-trapped excitonic state.

The observed relaxation processes in perylene film after one- or two-quantum excitation at 450 and 250 nm are summarized in Fig. 7. Triplet excitons are produced directly from upper excited electronic states with 108% quantum yield via singlet fission within < 30 fs. This ultrashort formation time is due to existence of large polycrystalline regions of H-aggregated perylenes and results from the strong coupling between upper singlet and triplet states and thus facilitating SF directly from S_2_ bypassing S_1_ state (violation of classical Kasha-Vavilov rule). Lifetime of triplet state is drastically shortened compared to crystal, as a result of efficient triplet–triplet annihilation. Competing processes are excimer formation and photoionization. Excimer is generated from singlet exciton within ~ 1 ps and subsequently emits yellow fluorescence. Photoionization results instantaneously after one- or two-quantum excitation (5–5.5 eV) with formation of dimeric radical cation. In addition, excitons are self-trapped due to a strong-field-induced lattice distortion through phonon-exciton coupling inside molecular packing. The lifetimes of self-trapped states are several hundreds of ps. Suppression of self-trapping and excimer formation by optimizing the film packing arrangement can be considered as the next step towards development of perylene based SF materials.

## Methods

### Film preparation and characterization

Perylene was purchased from Sigma-Aldrich and used as received. Films were prepared by thermal evaporation in vacuum evaporation pump device (QHV-Z350C, Pana Instruments), under low pressure of 10^–4^ Pa. The deposition rate was controlled at ~ 1.5 Å/s. Prior to deposition, the quartz substrates were cleaned with ethanol in an ultrasonic bath for 30 min and soaked in ethanol (12 h), water, acetone, ethanol (30 min each). The substrates were subsequently treated with plasma device (FEMTO SR CE, Diener) (0.3 mbar, 70 W) under O_3_ twice for 10 min.

X-ray diffraction (XRD) data of film were collected using a Rigaku D/Max-2400 X-ray diffractometer in parallel beam geometry employing Cu Kα line focused radiation at 9000 W (45 kV, 200 mA) power and equipped with a position sensitive detector with 10.0 nm radiation entrance slit. Samples were counted on zero background sample holders during the data acquisition. The best counting statistics were achieved by collecting samples using a 0.01° 2θ step scan from 5° to 80° with exposure time of 30 s per step.

Atomic Force Microscopy (AFM) was conducted on Tecnai F30 operated at 300 kV, HITACHI UHR FE-SEM SU8220 and Park Systems XE-70 with non-contact mode, respectively to characterize the surface structure and flatness of film.

The structure and morphology of film were investigated by use of field emission scanning electron microscope (FESEM, Nova Nanosem 450). SEM images were measured under 10 kV accelerating voltage and 10 µA current.

### Steady-state spectra

Absorption and fluorescence spectra were obtained by UV–visible spectrophotometer (Cary 100, Agilent) and spectrofluorometer (Fluorolog-3, Horiba Jobin Yvon), respectively.

### Time-resolved fluorescence spectra

Fluorescence lifetime measurements in sub-nanosecond range were carried out at room temperature by the time-correlated single photon counting (TCSPC) technique (PicoHarp 300, PicoQuant). By use of deconvolution/fit program (FluFit, PicoQuant) the time resolution was reached down to 10 ps. The second harmonic of a Ti-sapphire laser at 380 nm was used as the excitation source. Fluorescence lifetimes down to 100 fs were recorded by using up-conversion technique (TRFLS, Newport) in combination with the same laser (Mai Tai DeepSee, Spectra-Physics) (150 fs, 80 MHz)^9,55^.

Time-resolved PL spectra for longer decay times (450 ns) were measured using a fluorescence lifetime spectrometer (TemPro-01). Samples were excited at 389 and 450 nm with 1 MHz repetition rate.

### Femtosecond pump-probe spectra

Femtosecond transient absorption spectra were obtained by use of a home-made pump-probe setup^9,55^. Briefly, it consists of a mode-locked Ti–Sapphire amplified laser system (Spitfire Ace, Spectra-Physics). The output laser beam was at 800 nm with pulses width of 35 fs, repetition rate of 1 kHz and average power of 4 W. Pump beam (240–2400 nm) was generated through optical parametric amplifier (TOPAS, Light Conversion). The pump pulse duration was 30–40 fs (measured from the risetime of TA kinetics for rhodamine 6G). Probe beam, white light continuum (WLC), was generated in CaF_2_ rotating plate. The experimental data were fitted to a multiexponential decay function convoluted with the instrument response function; resulting time resolution was 20–30 fs. The pump beam diameter on the sample was 200 µm and 100 µm; power was 0.075 mW and 0.1 mW at 250 and 450 nm, respectively.

### Nanosecond flash photolysis

Nanosecond time-resolved transient absorption spectra were recorded on a LP980 laser flash photolysis spectrometer (Edinburgh Instruments Ltd.) in combination with a Nd:YAG laser (Surelite I-10, Continuum Electro-Optics, Inc.). Sample was excited with 355 nm laser pulse (1 Hz, 100 mJ per pulse, FWHM ≈ 7 ns).

### Global analysis

Global lifetime analysis of fsTA measurements were performed by Glotaran software^56^. The Decay Associated Spectra (DAS) allow separating several overlapping spectra. Parallel model was used for global analysis of time-resolved fluorescence and absorption spectra.

## Supplementary information

Supplementary information.
