# Supplementary material for: Ultrafast spectroscopy reveals singlet fission, ionization and excimer formation in perylene film
Source: Sci Rep. 2021 Mar 4;11:5220. doi: 10.1038/s41598-021-83791-z (PMC7933242; doi:10.1038/s41598-021-83791-z)
Supplement: Supplementary file 1 — Supplementary information. [file 41598_2021_83791_MOESM1_ESM.pdf]

Supplementary Information

Ultrafast spectroscopy reveals singlet fission, ionization and excimer formation in perylene film

Wenjun Ni[1], Licheng Sun[1,2], Gagik G. Gurzadyan[1]*

[1]State Key Laboratory of Fine Chemicals, Institute of Artificial Photosynthesis, Dalian University of Technology, Dalian116024, P.R. China.
[2]Department of Chemistry, School of Engineering Sciences in Chemistry, Biotechnology and Health, KTH Royal Institute of Technology, 10044 Stockholm, Sweden. email: gurzadyan@dlut.edu.cn; lichengs@kth.se

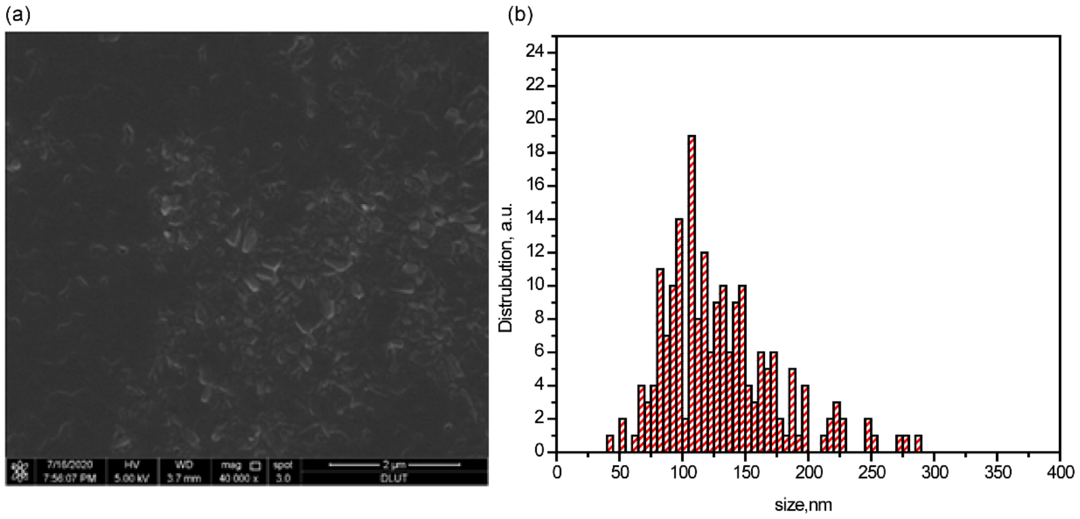

Figure S1. (a) The SEM image of perylene film and (b) the nanoaggregates size distribution from SEM measurements.

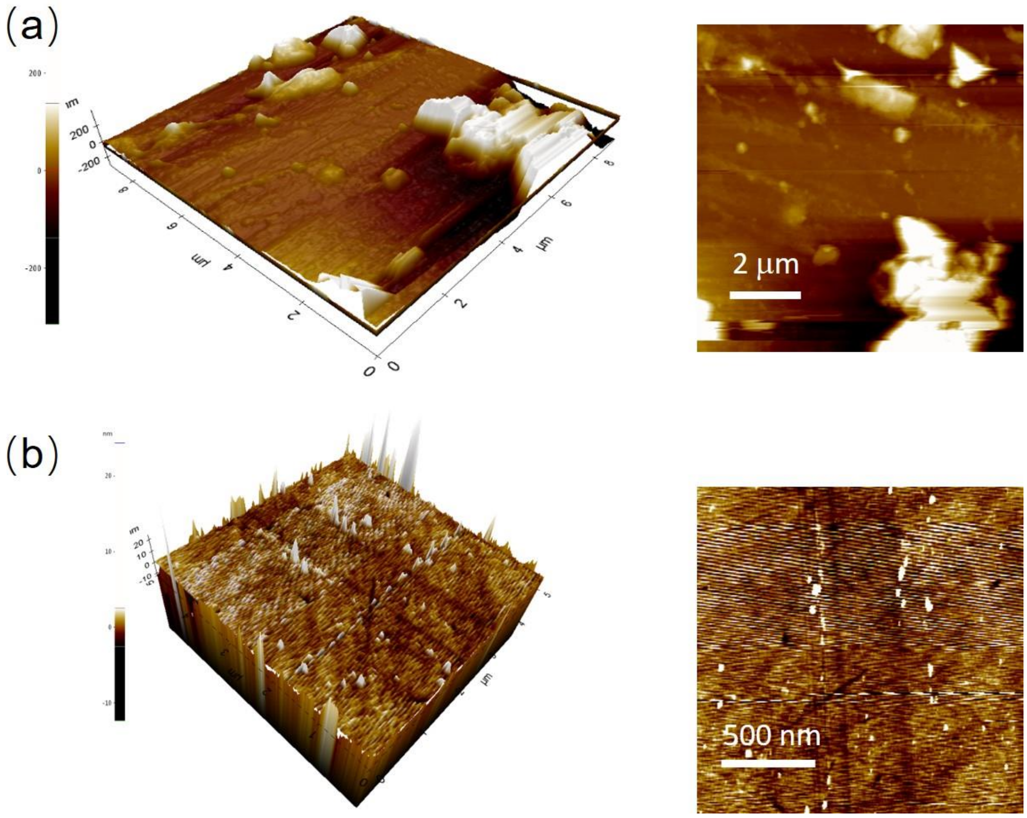

Figure S2. The 3D and 2D AFM images of perylene film: (a) 10 x 10 µm; (b) 2 x 2 µm

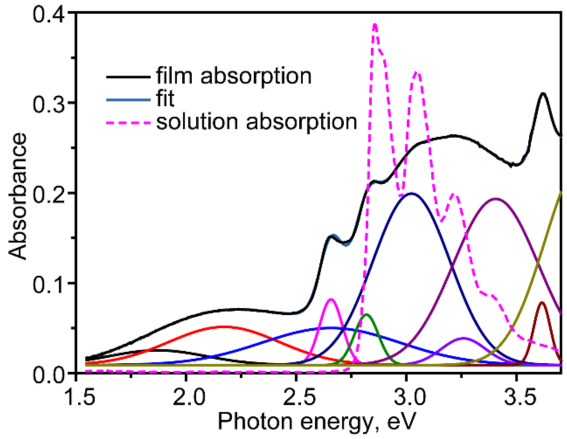

Figure S3. Gaussian multipeak fit of steady-state absorption spectrum of perylene film. Absorption spectrum of perylene in hexane is included as a reference.

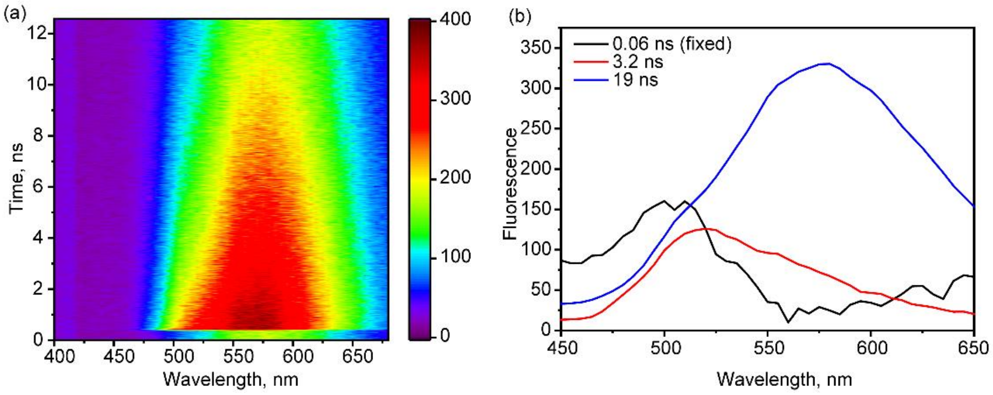

Figure S4. Time-resolved fluorescence map of perylene film, measured by TCSPC, λ_{ex} = 380 nm. (b) Global fit Decay-Associated Spectra (DAS) of TCSPC map.

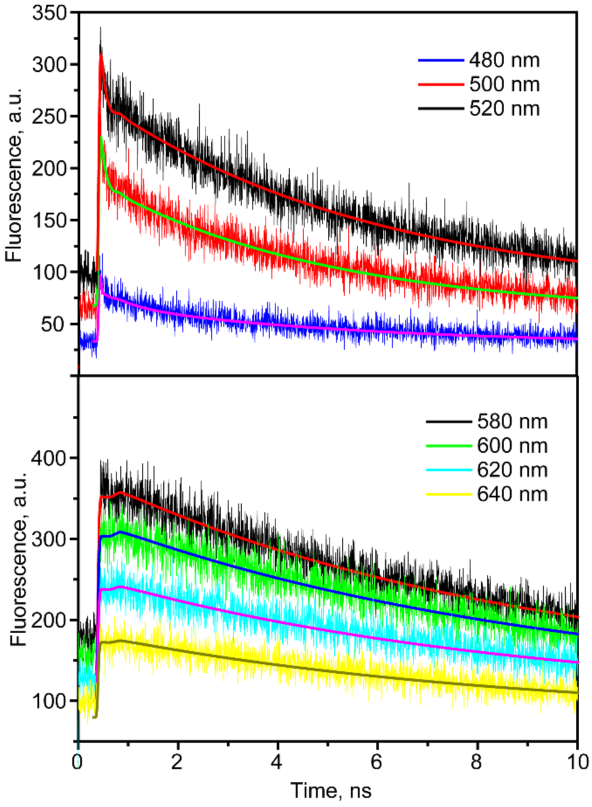

Figure S5. Time-resolved fluorescence kinetics in perylene film, λ_{ex} = 380 nm.

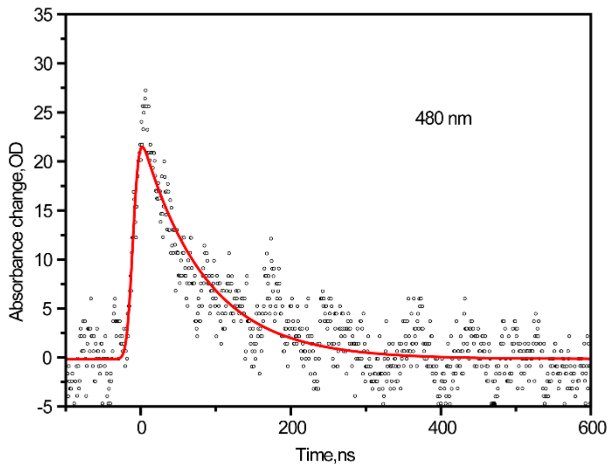

Figure S6. Nanosecond TA kinetics trace of perylene film at 480 nm, λ_{ex} = 355 nm.

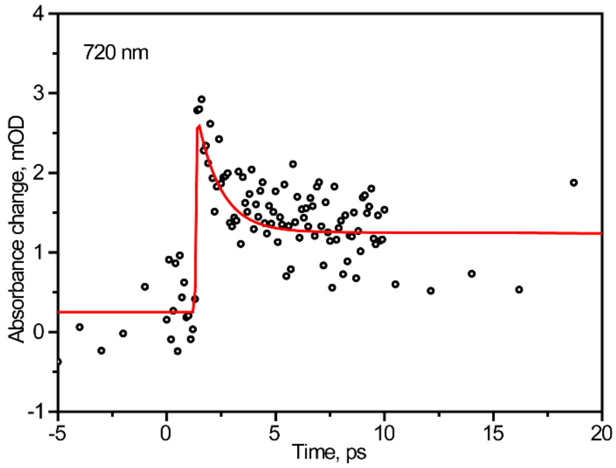

Figure S7. Femtosecond transient absorption kinetics of perylene film at 720 nm, λ_{ex} = 250 nm.

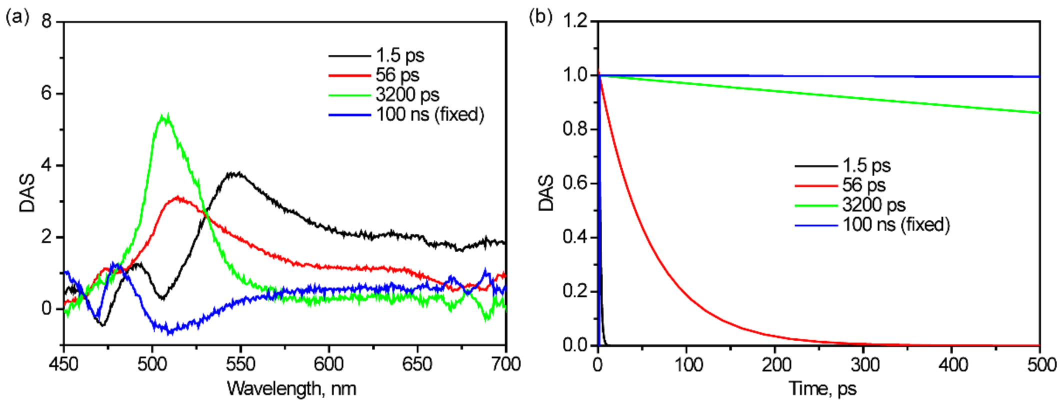

Figure S8. Global fit Decay-Associated Spectra (DAS) and kinetics of the fsTA at λ_{ex} = 250 nm of perylene film.

Table S1. Gaussian multipeak fit maxima of the steady-state absorption spectrum of perylene film; respective data for perylene in hexane is offered as a reference.

Film, eV	1.90	2.16	2.65	2.66	2.83	3.02	3.21	3.41	3.62
Solution, eV					2.85	3.04	3.23	3.43	

Table S2. Lifetimes obtained from fit/deconvolution of up-conversion results.

λ_{ex}, nm	λ_{probe}, nm	τ_1, ps	A_1	τ_2, ps	A_2
**400**	480	0.85±0.05	1		
	500	0.45±0.14	0.77	3.3±1.1	0.23
	520	1.0±0.06	0.98	100^f	0.02
	540	1.0±0.25	0.78	11±12	0.22
	560	0.74±0.17	0.64	11±4	0.36
	620			100^f	1

Table S3. Lifetimes obtained from fit/deconvolution of femtosecond transient absorption spectra above; f = fixed.

λ_{ex}, nm	λ_{probe}, nm	τ_1, ps	A_1	τ_2, ps	A_2	τ_3, ps	A_3
**250**	480	18±3	0.32	650±100	0.39	100000^f	0.30
	510	13±1.6	0.26	410±78	0.43	3300±700	0.31
	620	1.5±0.2	0.52	93±13	0.43	100000^f	0.06
	720	1.0±0.3	0.60			1200±430	0.40
**450**	480	0.17±0.03	-1	260±34	0.55	100000^f	0.45
	510			190±57	0.38	4500±930	0.62
	620			8.0±1.1	0.50	580±99	0.50
	720	0.2±0.1	0.42	4.9±1.2	0.29	1600±600	0.29

Nonlinear dependence fit [Ref. 1]

1) Two quantum absorption process:

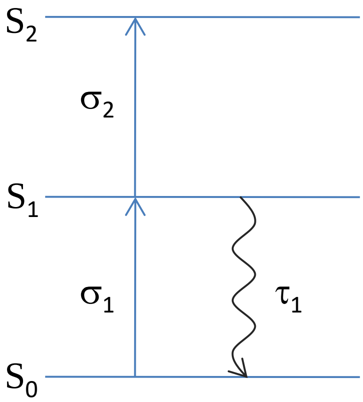

Schematic S1. Two-quantum absorption transitions

$$\frac{dN_0}{dt} = -\sigma_1 I N_0 + \frac{N_1}{\tau}$$

$$\frac{dN_1}{dt} = \sigma_1 I N_0 - \phi_2 \sigma_2 I N_1 - \frac{N_1}{\tau}$$

$$\frac{dN_2}{dt} = \phi_2 \sigma_2 I N_1$$

Where N_0, N_1, and N_2(TQA) are the populations of the ground state (S_0), intermediate state (S_1), and the two quantum excited state (S_2). σ_1 and σ_2 are the absorption cross sections of the $S_0 \rightarrow S_1$ and $S_1 \rightarrow S_2$ transitions, respectively (σ_1 = 9.2 x 10 $^{-17}$ cm^2 at 450 nm). ϕ_2 is the yield of the final product from the two quantum excited state. Analytical solution of the rate equations under assumption of a rectangular pulse shape and on the basis of $\tau_1 >> \tau_{pulse}$ (τ_1 is the lifetime of S_1 state):

$$N_2 = 1 - \frac{e^{-\sigma_1 E}}{1 - \frac{\sigma_1}{\varphi_2 \sigma_2}} - \frac{e^{-\varphi_2 \sigma_2 E}}{1 - \frac{\varphi_2 \sigma_2}{\sigma_1}}$$

Figure S9 shows dependence of the upper excited state population N_2 versus the intensity of the laser pulse at different pulse-widths and $\phi_2 \sigma_2$.

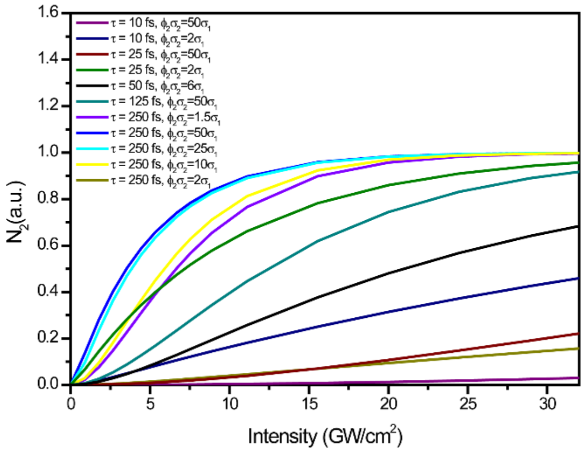

Figure S9. Population of TQA excited state N_2 versus the excitation laser intensity.

Singlet Fission Quantum yield

a) We used the triplet sensitization technique to estimate the molar extinction coefficient of triplet-triplet absorption of film. The specific dye (2,6-Diiodo-1,3,5,7-tetramethyl-8-phenyl-4,4-difluoroboradiazaindacene) (BODIPY2) was chosen as a sensitizer (Figure S10). The quantum yields of intersystem crossing and of triplet-triplet energy transfer from sensitizer to perylene are 97.3 and 100%, respectively.[2] We prepared the film with BODIPY and perylene mixture (molar ratio 1:1) by thermal evaporation. The sensitizer was excited at 550 nm. In addition, we took the standard sample benzophenone in acetonitrile (ϕ_T^{St} = 1.0) as a reference. Absorption spectra of reference molecule and perylene-BODIPY2 mixed film are presented in Figure S11.

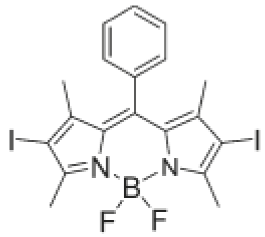

Figure S10. Chemical structure of sensitizer BODIPY2.

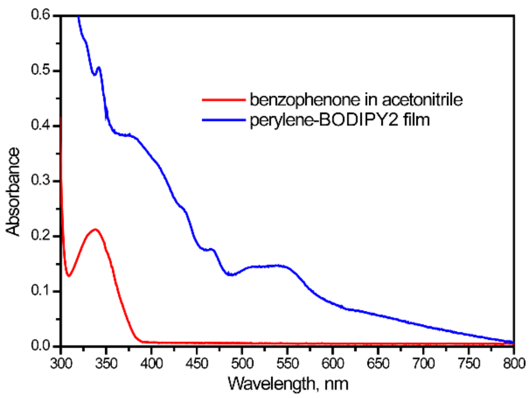

Figure S11. Absorption spectra of benzophenone in acetonitrile and of perylene-BODIPY2 film.

The triplet-triplet absorption molar extinction coefficient of film was calculated using the following equation[3-5]:

$$\varepsilon_T^X = \varepsilon_T^{S_t} \frac{OD_T^X}{OD_T^{S_t}} \frac{\phi_T^{S_t}}{\phi_T^X} \frac{P_{S_t}}{P_X} \frac{A_{S_t}}{A_X}$$

Where ϕ is the triplet quantum yield, OD are the optical densities of triplet absorption (obtained from TA), ε are the extinction coefficients of the triplet absorption, P is the power of the pump pulse and A is the absorbance at excitation wavelength. The subscript S_t refers to the standard molecule (benzophenone). X refers to perylene-BODIPY2 mixed film. The standard triplet absorption spectrum of benzophenone was obtained by pump-probe at λ_{ex} = 350 nm (Figure S12). Accordingly, the fs-TA spectra of perylene-BODIPY2 film is presented in Figure S13.

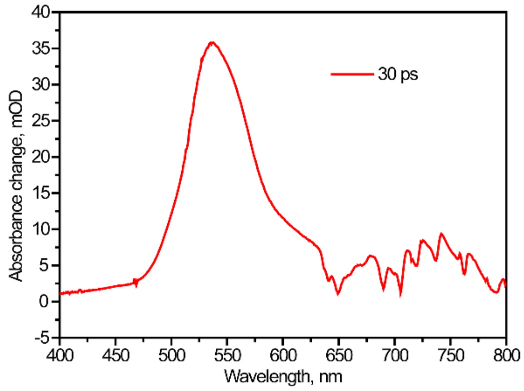

Figure S12. TA spectrum of triplet absorption of benzophenone in acetonitrile at λ_{ex} = 350 nm.

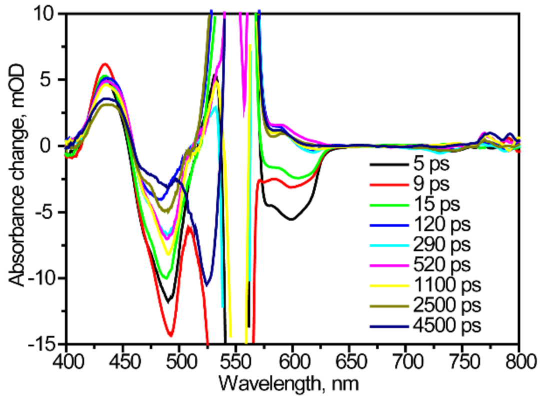

Figure S13. TA spectra of perylene-BODIPY2 film at λ_{ex} = 550 nm.

From TA spectra, we can get following information: ε_T^{St} = 6500 $M^{-1}cm^{-1}$,[6] OD_T^X = 5 mOD (Figure S13), $OD_T^{S_t}$ = 35 mOD (Figure S12), $\phi_T^{S_t}$= 1, ϕ_T^X= 1, P_{S_t}= 7.2x10^{-6} J/cm^2, P_X = 2.4x10^{-6} J/cm^2, A_{S_t}= 0.17 at λ_{ex} = 350 nm, A_X = 0.07 at λ_{ex} = 550 nm. Finally, we obtain the extinction coefficient ε_T^X (450 nm) = 6765 $M^{-1}cm^{-1}$. It should

be mentioned that large ground state bleaching and ESA from triplet of BODIPY2 are strongly distorting the TA spectrum and make it difficult obtaining the triplet extinction at the maximum (480 nm). Therefore, the obtained extinction coefficient of triplet absorption can be considered as the upper limit.

b) In order to avoid the strong GSB and ESA of BODIPY, anthracene with triplet energy 1.7 eV was chosen as a sensitizer. We prepared the film with anthracene and perylene mixture (molar ratio 1:1) by thermal evaporation. Anthracene is known as efficient triplet sensitizer for perylene. Absorption spectra of perylene-anthracene mixed film and the fs-TA spectra and kinetics of perylene-anthracene film are presented in the Figure S14 and S15, respectively.

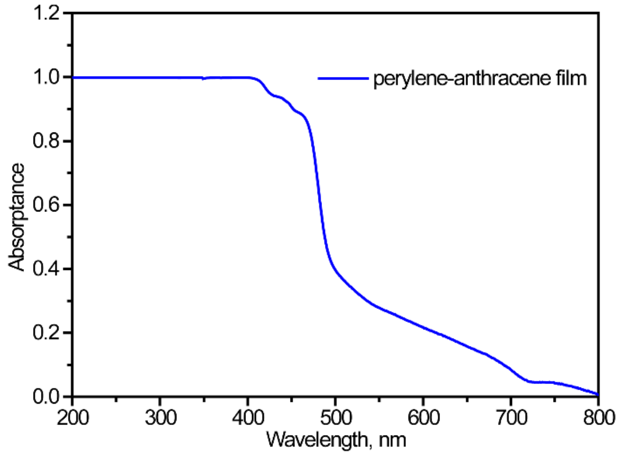

Figure S14. Absorption spectra of perylene-anthracene mixed film.

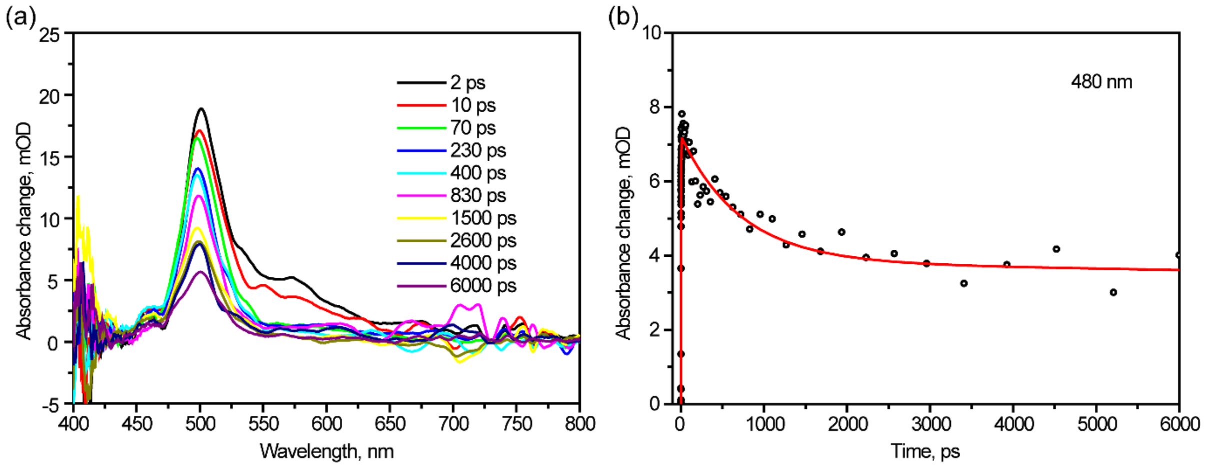

Figure S15. TA spectra (a) and triplet kinetics (b) of perylene-anthracene film at λ_{ex} = 350 nm.

From the spectra above, we can get the following information: ε_T^{St} = 6500 $M^{-1}cm^{-1}$,[6] OD_T^X = 4 mOD (Figure S15), OD_T^{St} = 35 mOD (Figure S12), ϕ_T^{St}= 1, ϕ_T^X= 1, P_{S_t}= 7.2x10^{-6} J/cm^2, P_X = 0.6 x10^{-6} J/cm^2, A_{S_t}= 0.17 at λ_{ex} = 350 nm, A_X = 1 at λ_{ex} = 350 nm. Finally, we obtain the extinction coefficient ε_T^X= 1082 $M^{-1}cm^{-1}$. It should be mentioned that there is some uncertainty in our calculations because at 350 nm both anthracene and perylene are absorbing. Therefore this value of extinction coefficient can be considered as the lower limit.

c) We used 3-bromoperylene with known quantum yield of intersystem crossing (ϕ_T^X= 0.12) and have prepared a film[7]. For calculations of the triplet absorption extinction coefficient we use the same equation as above. The subscript S_t refers to the standard molecule (benzophenone). X refers to 3-bromoperylene film (Figure S16). Absorption spectra and the fs-TA spectra and kinetics of 3-bromoperylene film are presented in the Figure S17 and S18, respectively.

$$\boldsymbol{\varepsilon_T^X = \varepsilon_T^{S_t} \frac{OD_T^X}{OD_T^{S_t}} \frac{\phi_T^{S_t}}{\phi_T^X} \frac{P_{S_t}}{P_X} \frac{A_{S_t}}{A_X}}$$

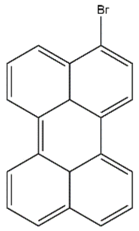

Figure S16. Chemical structure of 3-bromoperylene.

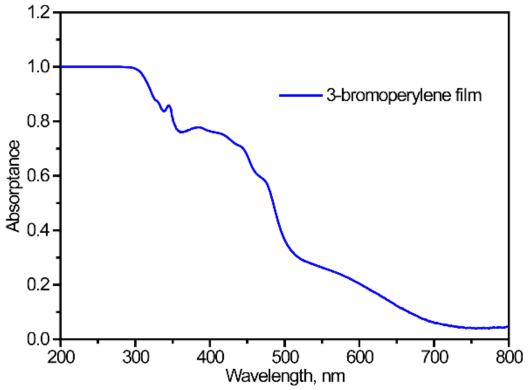

Figure S17. Absorption spectra of 3-bromoperylene film.

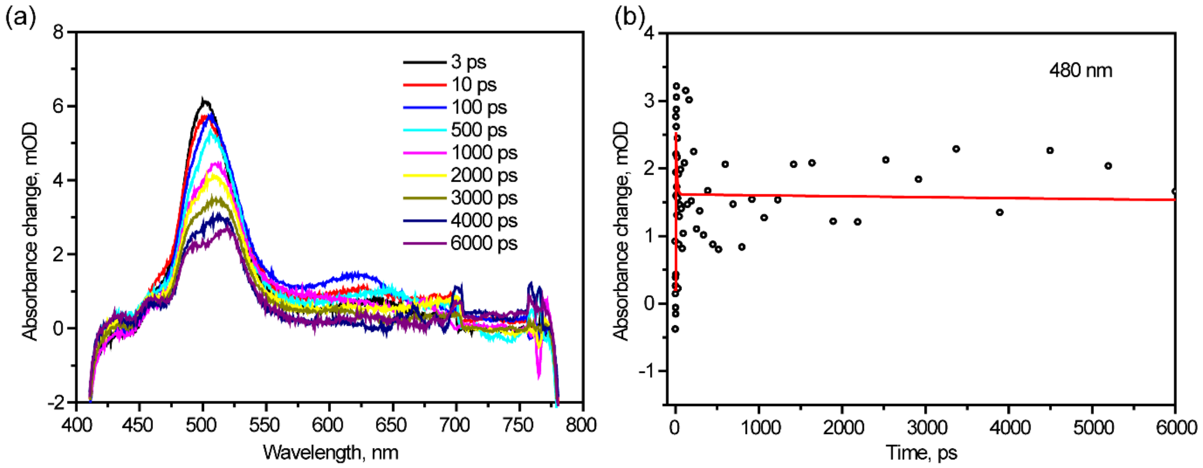

Figure S18. TA spectra (a) and triplet kinetics (b) of 3-bromoperylene film at λ_{ex} = 400 nm.

From the spectra above, we can get the following information: ε_T^{St} = 6500 $M^{-1}cm^{-1}$, OD_T^X = 1.7 mOD (Figure S19), OD_T^{St} = 35 mOD (Figure S12), ϕ_T^{St} = 1, ϕ_T^X = 0.12, P_{S_t} = 7.2x10^{-6} J/cm^2, P_X = 1.2x10^{-6} J/cm^2, A_{S_t} = 0.17 at λ_{ex} = 350 nm, A_X = 0.76 at λ_{ex} = 400 nm. Finally, we obtain the extinction coefficient ε_T^X = 3531 $M^{-1}cm^{-1}$.

Uncertainty of the results obtained from first two methods (a and b), i.e. by using BODIPY and anthracene as sensitizers, is too big (see above). Therefore the calculations of the quantum yield of triplet formation (accordingly, of singlet fission) we perform only by comparison with 3-bromoperylene (c). We use the following equation:

$$\boldsymbol{\phi_T^X = \phi_T^{S_t} \frac{OD_T^X}{OD_T^{S_t}} \frac{\varepsilon_T^{S_t}}{\varepsilon_T^X} \frac{P_{S_t}}{P_X} \frac{A_{S_t}}{A_X}}$$

Where ϕ is the triplet quantum yield, OD are the optical densities of triplet absorption (obtained from TA), ε are the extinction coefficients of the triplet absorption, P is the power of the pump pulse and A is the absorbance (or absorptance, in the case of strong absorption) at excitation wavelength. The subscript S_t refers to the reference 3-bromoperylene film. X refers to perylene film. The triplet absorption TA spectra of 3-

bromoperylene film were obtained by pump-probe at λ_{ex} = 350 nm (Figure S19). Accordingly, the fs-TA spectra of perylene film are presented in Figure 4.

From TA spectra, we can get the following information: $\phi_T^{S_t}$= 0.12, OD_T^X = 3.5 mOD (Figure 4), $OD_T^{S_t}$ = 1.7 mOD (Figure S19), ε_T^{St} = ε_T^X = 3531 $M^{-1}cm^{-1}$, P_{S_t}= 1.2x10^{-6} J/cm^2, P_X = 0.23x10^{-6} J/cm^2, A_{S_t}= 0.76 at λ_{ex} = 400 nm, A_X = 0.93 at λ_{ex} = 250 nm. The quantum yield of triplet state is 108%. Accordingly, the singlet fission yield is 54%.

References

1. Ma, L., Tan, K. J., Jiang, H., Kloc, C., Michel-Beyerle, M.-E. & Gurzadyan, G. G. Excited-state dynamics in an alpha-perylene single crystal: two-photon- and consecutive two-quantum-induced singlet fission. *J. Phys. Chem. A* **118**, 838-843 (2014).
2. Wu, W., Guo, H., Wu, W., Ji, S. & Zhao, J. Organic Triplet Sensitizer Library Derived from a Single Chromophore (BODIPY) with Long-Lived Triplet Excited State for Triplet-Triplet Annihilation Based Upconversion. *J. Org. Chem.* **76**, 7056-7064 (2011).
3. Korovina, N. V., Chang, C. H. & Johnson, J. C. Spatial separation of triplet excitons drives endothermic singlet fission. *Nature Chem.* **12**, 391 (2020).
4. Roberts, S. T., McAnally, R. E., Mastron, J. N., Webber, D. H., Whited, M. T., Brutchey, R. L., Thompson, M. E. & Bradforth, S. E. Efficient Singlet Fission Discovered in a Disordered Acene Film. *J. Am. Chem. Soc.* **134**, 6388-6400 (2012).
5. Truscott, T. G., Pulse Radiolysis and Flash Photolysis. *Photobiology: The Science and Its Applications*, Springer US: Boston, ISBN: 978-1-4613-6661-4 (1991).
6. Carmichael, I., Helman, W. P.& Hug, G. L., Extinction Coefficients of Triplet–Triplet Absorption Spectra of Organic Molecules in Condensed Phases: A Least-Squares Analysis. *J. Phys. Chem. Ref. Data* **16**, 239-260 (1987).
7. Lewitzka, F., Lohmannsroben, H. G., Strauch, M. & Luttke, W. Photophysical Properties of Methyiperylenes and Bromoperylenes in Solution. *J. Photochem. Photobiol. A* **61**, 191-200 (1991).
